# Supplementary material for: The Role of Metal Nanoparticles in the Pathogenesis of Stone Formation
Source: Int J Mol Sci. 2024 Sep 5;25(17):9609. doi: 10.3390/ijms25179609 (PMC11394863; doi:10.3390/ijms25179609)
Supplement: Supplementary file 1 [file ijms-25-09609-s001.zip › Supplementary S2.pdf]

Supplementary Figure S1.

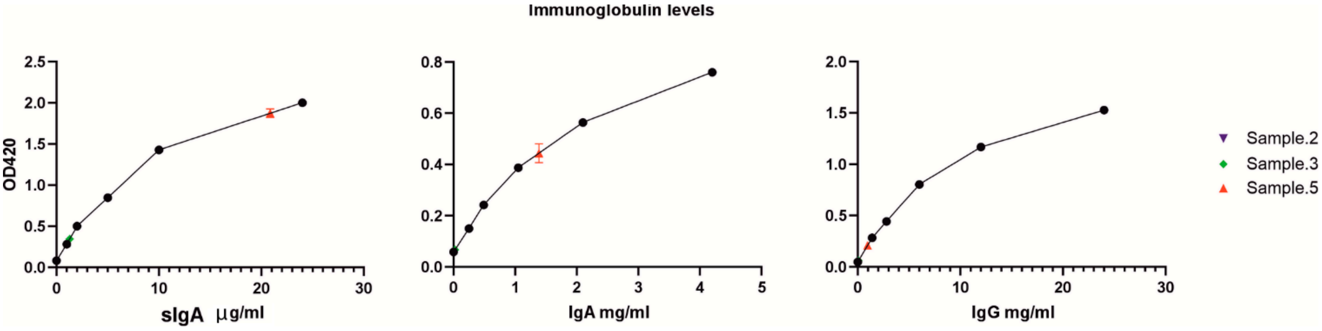

Figure S1. Adsorptions at OD_{420} of immunoglobulins concentrations of the calibration series from the appropriateassay kits, and optical densities of the analyzed samples fitted to a point-to-point curve.

Supplementary Table S1. Limits of quantification*.

#	IgA_s	IgG	IgA
Concentration (µg/ml)	0.35	200.0	21.4

* According to the manufacturer's instructions.

Supplementary Table S2. Initial concentration of immunoglobulins in the supernatant dilutions.

Sample	IgG, (µg/ml)	IgA, (µg/ml)	Secretory IgA, (µg/ml)
Sample 2	not available	not available	not available
Sample 3	not available	24.457	1.288
Sample 5	960.256	1288.136	20.866

Supplementary Table S3. Concertation (corrected dilution).

Sample	IgG, (µg/ml)	IgA, (µg/ml)	Secretory IgA, (µg/ml)	Total protein, (µg/ml)
Sample 2	not available	not available	not available	308
Sample 3	not available	0.098	0.052	216
Sample 5	3.841	5.553	0.835	524
